# Sea level variability and modeling in the Gulf of Guinea using supervised machine learning

**DOI:** 10.1038/s41598-023-48624-1

**Published:** 2023-12-03

**Authors:** Akeem Shola Ayinde, Huaming Yu, Kejian Wu

**Affiliations:** 1 College of Oceanic and Atmospheric Sciences, Ocean University of China, Qingdao, 266100 China; 2 Physical Oceanography Laboratory, Ocean University of China, Qingdao, 266100 China; 3 Department of Marine Meteorology and Climate, Nigerian Institute for Oceanography and Marine Research, PMB 12729, Victoria Island, Lagos Nigeria

**Keywords:** Ocean sciences, Physical oceanography

## Abstract

The rising sea levels due to climate change are a significant concern, particularly for vulnerable, low-lying coastal regions like the Gulf of Guinea (GoG). To effectively address this issue, it is crucial to gain a comprehensive understanding of historical sea level variability, and the influencing factors, and develop a reliable modeling system for future projections. This knowledge is essential for informed planning and mitigation strategies aimed at protecting coastal communities and ecosystems. This study presents a comprehensive analysis of mean sea level anomaly (MSLA) trends in the GoG between 1993 and 2020, covering three distinct periods (1993–2002, 2003–2012, and 2013–2020). It investigates the connections between interannual sea level variability and large-scale oceanic and atmospheric forcings. Furthermore, the study evaluates the performance of supervised machine learning techniques to optimize sea level modeling. The findings reveal a consistent rise in MSLA linear trends across the basin, particularly pronounced in the northern region, with a total linear trend of 88 mm over the entire period. The highest decadal trend (38.7 mm) emerged during 2013–2020, with the most substantial percentage increment (100%) occurring in 2003–2012. Spatial variation in decadal sea-level trends was influenced by subbasin physical forcings. Strong interannual signals in the spatial sea level distribution were identified, linked to large-scale oceanic and atmospheric phenomena. Seasonal variations in sea level trends are attributed to seasonal changes in the forcing factors. The evaluation of supervised learning modeling methods indicates that Random Forest Regression and Gradient Boosting Machines are the most accurate, reproducing interannual sea level patterns in the GoG with 97% and 96% accuracy. These models could be used to derive regional sea level projections via downscaling of climate models. These findings provide essential insights for effective coastal management and climate adaptation strategies in the GoG.

## Introduction

Climate change is a pressing global concern, with its escalating impacts significantly affecting sea levels^1^. By comprehending and modeling sea level trends, we can gain crucial insights into how climate change is impacting coastal regions, especially in the Gulf of Guinea, a wide expanse of land on the West African coast, where coastlines are predominantly low-lying. Therefore, there is an urgent need for sea level projection models to assess the potential impacts of sea level rise. These models are invaluable for policymakers and coastal planners, enabling them to proactively prepare for and mitigate the consequences on coastal communities and ecosystems. The examination of regional sea level variability and the identification of its driving factors are of paramount importance in understanding the consequences of climate change on coastal areas^2^. The range of sea level variability encompasses a wide spectrum of challenges, with profound implications for coastal communities, infrastructure, and marine ecosystems. These challenges encompass elevated storm surges, coastal erosion, flooding, saltwater intrusion, disruption of marine ecosystems, and infrastructure damage, all of which carry substantial economic implications. The intensification of these impacts, particularly in low-lying coastal regions, underscores the critical need for comprehending the mechanisms underlying sea level variability and for establishing precise, cost-effective models to elucidate regional sea level drivers and projections.

Conventional numerical models, including deterministic numerical models which are based on mathematical equations that describe the physical processes governing a system (such as dynamical or physical models), have a long history of demonstrating their effectiveness in forecasting variations in sea state and sea level^3–5^. Nevertheless, the practical deployment and execution of these models can entail substantial intricacies and expenses, meaning that they come with challenges and costs when it comes to implementing and using them in real-world applications. Lately, the emergence of machine learning techniques has offered promising avenues to enhance the prediction and forecasting capabilities of sea levels. These advancements extend beyond predictions and forecasts, with machine learning showcasing its potential in refining 'best-estimate' ensemble forecasts for ocean waves^6^ and predicting storm surges with precision^7^. Particularly, the performance of ANN matches that of deterministic hydrodynamic models in capturing extreme events. In recent times, ANN has been successfully employed for storm surge hindcasts in estuarine ports in the UK, enabling accurate coastal flood predictions^8^. Furthermore, the predictive aptitude of ANN extends to oceanic variables such as subsurface temperature (ST) and even climate phenomena like El Niño/Southern Oscillation (ENSO) over 1.5 years^9^. Impressively, ANN surpasses state-of-the-art dynamical forecast systems in forecasting ENSO, highlighting remarkable advancements in ENSO predictions^10^.

The ascendant integration of Artificial Intelligence (AI) in the scientific realm has necessitated the development of diverse machine learning approaches, encompassing Convolutional Neural Networks (CNN), Recurrent Neural Networks (RNN), Feedforward Neural Networks (FNN), and traditional regression methods. While CNNs excel in image recognition, object detection, and classification tasks^11–13^, RNNs are well-suited for temporal analyses, natural language processing, and speech recognition^14,15^. In contrast, FNNs are adept at pattern recognition, function approximation, and mapping input features to output targets. The efficacy of the machine learning models hinges upon data quality, quantity, and a solid understanding of the system to discern requisite input data (predictors/training data) for the analysis at hand. Marine meteorological data have been particularly successful as input variables, demonstrating effectiveness in modeling and forecasting sea level variability^16–18^.

This study examines the spatial linear trend of sea levels and its drivers in the GoG, focusing on decadal trends. Additionally, it investigates seasonal variability and the impact of large-scale oceanic and atmospheric phenomena on interannual sea level fluctuations. We develop and evaluate five supervised machine learning models, including two artificial neural networks (ANN): Multi-layer Perceptron Regression (MLPR), which is an FNN, and Long Short-Term Memory (LSTM), an example of RNN. We also employ traditional regression models: Multiple Linear Regression (MLR), Random Forest Regression (RFR), and Gradient Boosting Machine (GBM). These models utilize marine meteorological and hydrological variables, including thermosteric sea level anomaly (TSLA), halosteric sea level anomaly (HSLA), wind stress curl (WSC), atmospheric pressure, net heat flux (NHF), precipitation, evaporation, and freshwater runoff. These variables have been widely recognized and studied in previous research, and their influence on sea level fluctuations is well-documented^19–21^. The manuscript is structured as follows: Section "Results" introduces the study area, data sources, and variable parameterization. In Section "Discussion/conclusion", we detail the methodology and model specifications. Subsequently, in Section "Methodology", we present and discuss the experimental outcomes.

## Results

### Interannual-to-interdecadal spatial trends and variability of MSLA and its forcings

In this section, we analyzed the interannual-to-interdecadal spatial trends and variability of MSLA and its associated drivers, including SSLA, TSLA, HSLA, ocean heat content (OHC), WSC, air temperature, NHF, precipitation, evaporation, freshwater runoff, and atmospheric pressure. We aimed to investigate their potential contributions to MSLA during the period from 1993 to 2020 in the GoG. Subsequently, we compared the spatial trends and variability for the three distinct periods: 1993–2002, 2003–2012, and 2013–2020. This division was based on the need to capture and analyze long-term trends while avoiding the potential masking of significant shorter-term variability. This approach allows us to distinguish between gradual, sustained changes and shorter-term variations, and to assess how sea level variability and its drivers have evolved over time. In general, analysis of MSLA variability revealed a distinct spatial and temporal pattern across the basin, which was significantly influenced by subbasin-scale drivers.

### Linear trends during 1993–2002

**Figure 1:**
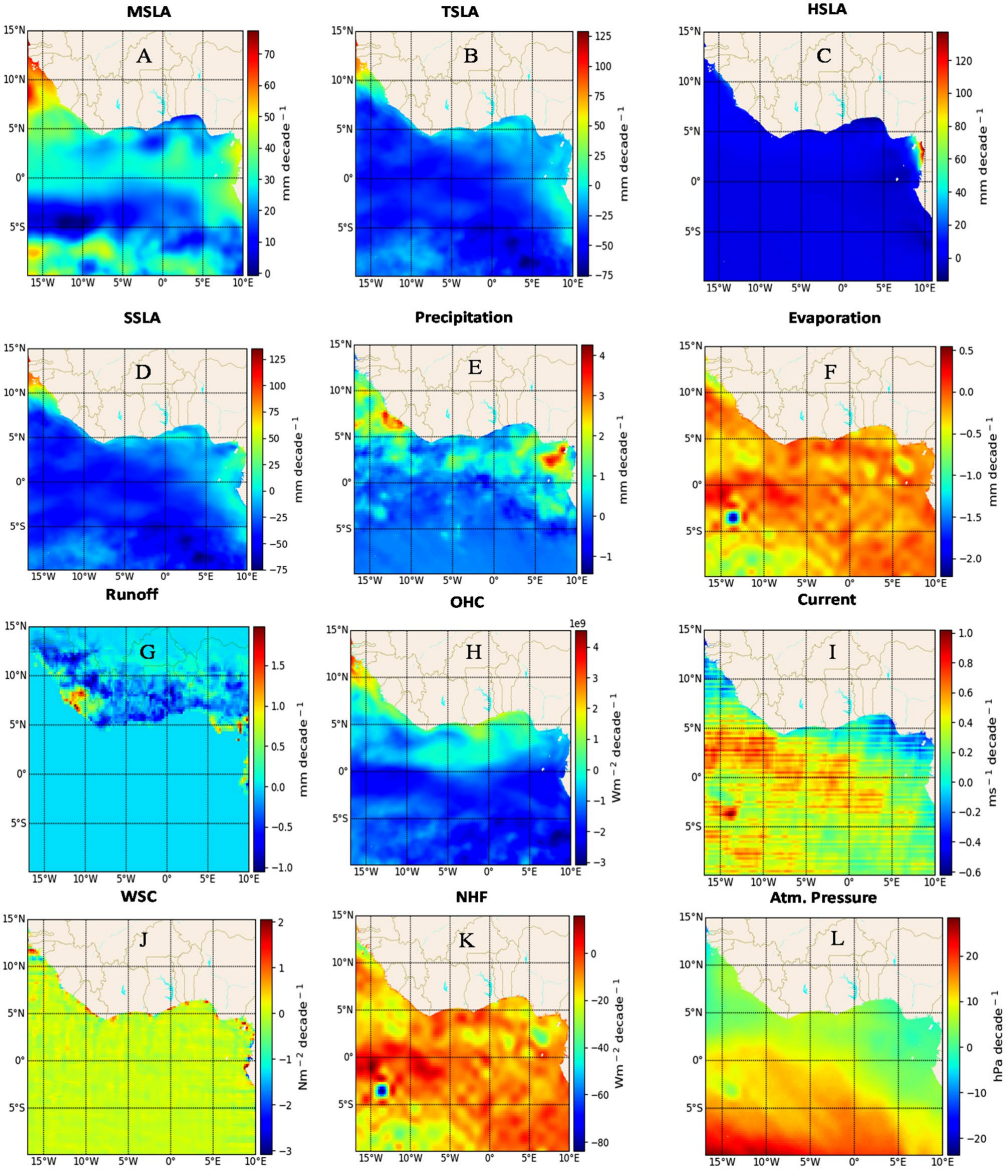
The spatial linear trend of mean sea level anomaly (MSLA) (**A**) and its forcing factors, including steric sea level anomaly (SSLA) (**B**), thermosteric sea level anomaly (TSLA) (**C**), halosteric sea level anomaly (HSLA) (**D**), precipitation (**E**), evaporation (**F**), runoff (**G**) ocean heat content (OHC) (**H**), Current (**I**), wind stress curl (WSC) (**J**), net heat flux (NHF) (**K**), and atmospheric pressure (**L**) for the period 1993 to 2002 in the Gulf of Guinea. The trends were obtained by subjecting the monthly mean of each dataset to a linear regression model, which calculates decadal slope at each grid point. The calculations, as well as the maps, were produced with a Python (3.10.9) script.

The spatial distribution of linear trends in MSLA from 1993 to 2002 reveals notable variations in trends ($11.8\,\mathrm{ mm }\,{\mathrm{decade}}^{-1}$) across the GoG basin (Fig. 1A). Substantial trends are evident along the northern coast, particularly pronounced ($\sim 55{-}60\,\mathrm{ mm }\,{\mathrm{decade}}^{-1}$) along Sierra Leone, Guinea Conakry, Guinea Bissau, Gambia, and Senegal. Comparatively higher trends are observed along the continental shelf, except for Nigeria, Benin, Togo, Ghana, Gabon, and Congo, which exhibit lower trends. The variability in these spatial trends is closely tied to the underlying sub-basin forcing factors. TSLA's basin-wide negative trend (-$19\,\mathrm{ mm }\,{\mathrm{decade}}^{-1}$) mirrors MSLA's coastal trend (Fig. 1A,C), while HSLA's basin-wide positive trend (0.6 $\mathrm{mm }\,{\mathrm{decade}}^{-1}$) exhibits pronounced variability on the northeast coast (Fig. 1D). Both TSLA and HSLA contribute to SSLA's overall spatial trend (-$18.6\,\mathrm{ mm }\,{\mathrm{decade}}^{-1}$), aligning closely with MSLA's trend along the northern coast (Fig. 1A–D). Notably, regions experiencing decreased evaporation trends coincide with increased MSLA trends, while areas with high (low) runoff and precipitation correspond to regions with high (low) MSLA, and vice versa (Fig. 1A,E,F,G). While there is no significant change in the WSC trend offshore, cyclonic WSC trends dominate the northern basin, influencing MSLA variations (Fig. 1J). Negative current velocity trends are linked with cyclonic WSC trends, leading to low MSLA, except along the Gambia coast, where high MSLA is observed (Fig. 1A,I,J). The role of cyclonic WSC in coastal upwelling and MSLA modulation is well-documented^22–24^. Atmospheric pressure shows a strong spatial trend (4.9 $\mathrm{hPa }\,{\mathrm{decade}}^{-1}$), decreasing meridionally from the south to north basin. High atmospheric pressure in the south drives northward flow, raising (lowering) water levels respectively (Fig. 1A,L). The NHF's spatial linear trend is negative ($-7.2\,{\mathrm{Wm}}^{-2}\,{\mathrm{decade}}^{-1}$), signifying ocean heat release into the atmosphere and a net heat loss. This contributes to decreasing OHC ($-8.6 \times{ 10}^{8}\,{\mathrm{Wm}}^{-2}\,{\mathrm{decade}}^{-1}$), leading to a lower MSLA trend (Fig. 1A,H,K).

### Linear trends during 2003–2012

**Figure 2:**
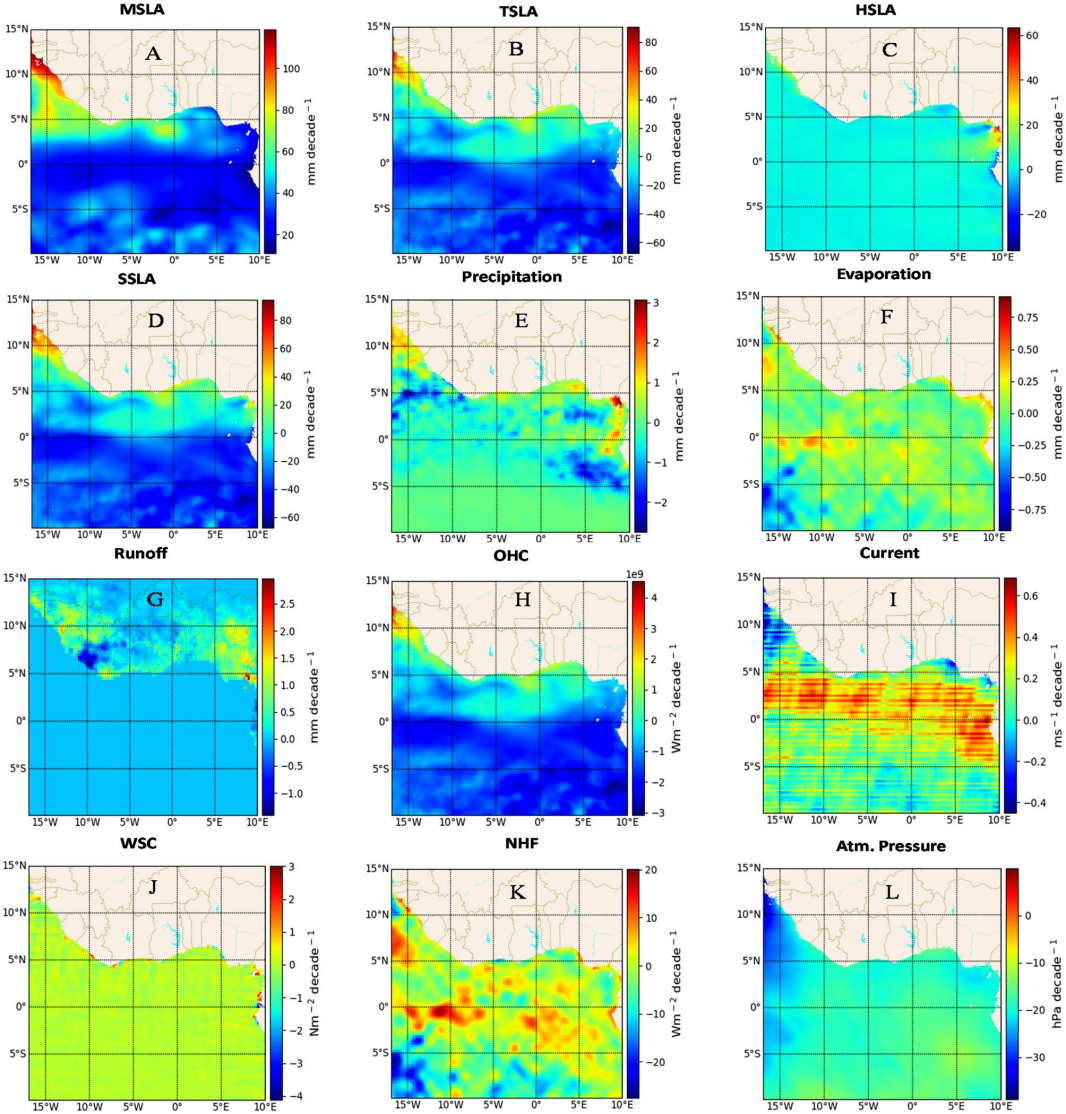
The spatial linear trend of mean sea level anomaly (MSLA) (**A**) and its forcing factors, including steric sea level anomaly (SSLA) (**B**), thermosteric sea level anomaly (TSLA) (**C**), halosteric sea level anomaly (HSLA) (**D**), precipitation (**E**), evaporation (**F**), runoff (**G**) ocean heat content (OHC) (**H**), Current (**I**), wind stress curl (WSC) (**J**), net heat flux (NHF) (**K**), and atmospheric pressure (**L**) for the period 2003 to 2012 in the Gulf of Guinea. The trends were obtained by subjecting the monthly mean of each dataset to a linear regression model, which calculates decadal slope at each grid point. The calculations, as well as the maps, were produced with a Python (3.10.9) script.

The period from 2003 to 2012 witnessed distinctive changes in the spatial distribution of linear trends in MSLA and its driving forces, compared to 1993–2002, with heightened sea level trends in the northwestern basin (Fig. 2A). Specifically, the northwest basin along the coasts of Guinea Conakry, Guinea Bissau, Gambia, and Senegal experienced the highest trends, at approximately $100\,\mathrm{ mm }\,{\mathrm{decade}}^{-1}$, while the northeast basin and offshore areas showed lower trends. In general, MSLA exhibited a remarkable increase in spatial linear trend, with a value of $23.5\,\mathrm{ mm }\,{\mathrm{decade}}^{-1}$, constituting a 100% surge from the previous decade in the GoG. Similarly, TSLA and HSLA displayed overall increased spatial linear trends, with values of $-16.3\,\mathrm{ mm }\,{\mathrm{decade}}^{-1}$ decade and $0.81\,\mathrm{ mm }\,{\mathrm{decade}}^{-1}$, respectively, with varying magnitudes across subbasins (Fig. 2C,D). These trends notably contributed to SSLA and MSLA at the coasts (Fig. 2A,B). Hydrological variables, including precipitation, runoff, and evaporation, demonstrated consistent spatial linear trend patterns, which had a similar impact on MSLA compared to the previous decade. This was particularly evident along northern coasts, where high trends in these variables correlated with MSLA trends. However, an exception was observed on the Nigerian coast, where a higher evaporation trend led to lower HSLA and MSLA (Fig. 2A,D–G). The spatial linear trend of WSC and current velocities resembled the previous decade's pattern but with slightly lower overall linear trends ($0.004 \,{\mathrm{Nm}}^{-2}\,{\mathrm{decade}}^{-1}$ and $0.2 \,{\mathrm{ms}}^{-1}\,{\mathrm{decade}}^{-1}$ respectively). Conversely, atmospheric pressure showed a unique pattern, characterized by a decreased spatial linear trend of − 16.5 hPa, influencing MSLA in areas like Guinea Conakry, Guinea Bissau, Gambia, and Senegal (Fig. 2A,L). Low linear trends in WSC, current velocity, and atmospheric pressure negatively correlated with sea level, enhancing MSLA (Fig. 2A,I,J,L). Despite a negative NHF spatial linear trend ($-1.007 \,{\mathrm{Wm}}^{-2}\,{\mathrm{decade}}^{-1}$), it represented an 86% increase from the prior decade, impacting OHC trends ($-7.5 \times{ 10}^{8} \,{\mathrm{Wm}}^{-2}\,{\mathrm{decade}}^{-1}$) and further amplifying SSLA and MSLA trends during this period (Fig. 2A,B,H,K).

### Linear trends during 2013–2020

**Figure 3:**
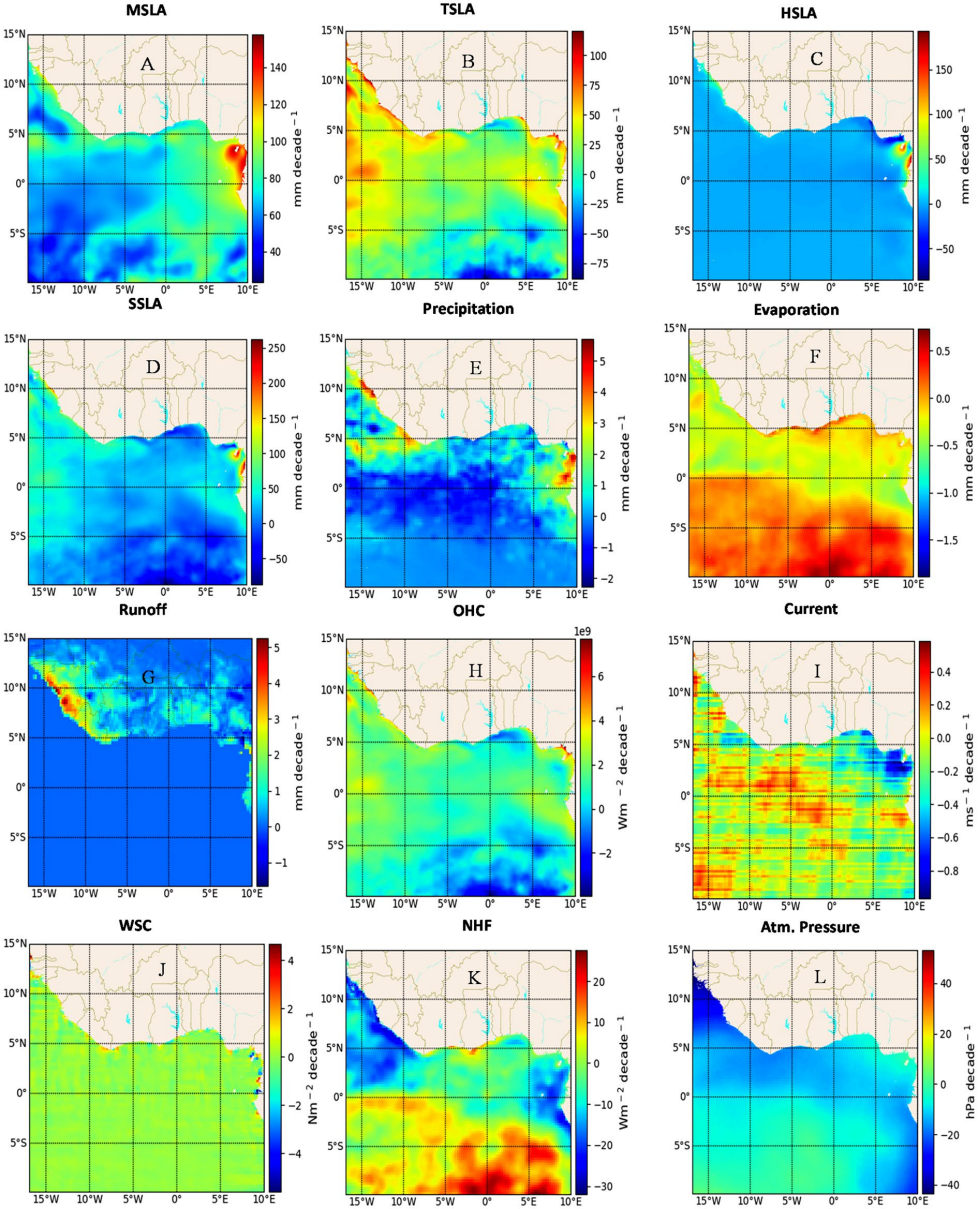
The spatial linear trend of mean sea level anomaly (MSLA) (**A**) and its forcing factors, including steric sea level anomaly (SSLA) (**B**), thermosteric sea level anomaly (TSLA) (**C**), halosteric sea level anomaly (HSLA) (**D**), precipitation (**E**), evaporation (**F**), runoff (**G**) ocean heat content (OHC) (**H**), Current (**I**), wind stress curl (WSC) (**J**), net heat flux (NHF) (**K**), and atmospheric pressure (**L**) for the period 2013 to 2020 in the Gulf of Guinea. The trends were obtained by subjecting the monthly mean of each dataset to a linear regression model, which calculates decadal slope at each grid point. The calculations, as well as the maps, were produced with a Python (3.10.9) script.

In the period spanning 2013 to 2020, the behavior of MSLA diverges from the previous two decades, showcasing distinct spatial trends and magnitudes with elevated sea level trends in the eastern basin (Fig. 3A). Notably, a considerable upsurge in MSLA is observed across the basin, with a spatial linear trend of $31.7\,\mathrm{ mm }\,{\mathrm{decade}}^{-1}$. This trend marks a substantial increment of about 169% in comparison to the 1993–2002 periods, and a 32% increase from the 2003–2012 spans. The eastern basin stands out with a notable linear trend, particularly evident along the coasts of Cameroon and Equatorial Guinea. However, the trend in HSLA presents a contrasting scenario, experiencing a significant decrease (-$0.43\,\mathrm{ mm }\,{\mathrm{decade}}^{-1}$) in comparison to the previous two decades (Fig. 3D). This reduction amounts to approximately 28% from the first decade and 47% from the second, yet the distribution of spatial trends remains akin to that of MSLA. This decrease in HSLA predominantly stems from negative trends along specific coasts, particularly the Nigerian coast, a result of diminishing freshwater runoff and heightened evaporation in these areas. Meanwhile, TSLA displays an overall positive spatial linear trend of $13.6\,\mathrm{ mm }\,{\mathrm{decade}}^{-1}$, reflecting substantial increments of 183% and 172% from the preceding decades, respectively (Fig. 3C). However, the spatial pattern of TSLA slightly diverges from MSLA during this timeframe, with the highest trend observed in the western basin and particularly the northwestern shelf. The combined spatial trends of HSLA and TSLA significantly contribute to the spatial linear trend of SSLA (Fig. 3B–D), reaching a peak of $14.1\,\mathrm{ mm }\,{\mathrm{decade}}^{-1}$. This indicates an impressive increment of 191% and 175% in comparison to the previous decades, respectively. The linear trends of precipitation and runoff display a consistent pattern along the coast, counterbalancing evaporation, with the northern basin exhibiting a high precipitation trend corresponding to low evaporation in the southern basin (Fig. 3E–G). During this period, the spatial linear trend of both WSC ($0.44\,\mathrm{ mm }\,{\mathrm{decade}}^{-1}$), and current velocity ($-0.07 \,{\mathrm{ms}}^{-1}\,{\mathrm{decade}}^{-1}$) displays a negative trend, representing a decrease of about 300% for WSC and 158% for current velocity compared to the previous decade (Fig. 3I,J). Similarly, atmospheric pressure exhibits a negative trend of ($-5.5\,\mathrm{ hPa }\,{\mathrm{decade}}^{-1}$), signifying a 67% increase from the last decade, with a distinct distribution along the coasts and offshore regions (Fig. 3L). The spatial linear trend of NHF portrays a positive trend ($0.008 \,{\mathrm{Wm}}^{-2}\,{\mathrm{decade}}^{-1}$), indicating an increase of 108% from the previous decade (Fig. 3K). Similarly, OHC demonstrates a positive trend of $6.6 \times{ 10}^{8}\, {\mathrm{Wm}}^{-2}\,{\mathrm{decade}}^{-1}$, corresponding to a remarkable 188% increment (Fig. 3H). These affirmative trends contribute to the observed heightened SSLA and MSLA during this period. The summarized analysis is presented in Table 1.

**Table 1:** Summary of the magnitude and percentage changes in the trends of MSLA and its forcing factors for the periods 1993–2002, 2003- 2012, 2013–2020, and 1993–2020 in the GoG.

Variable	Period /trend			%Changes		
	1993–2002	2003–2012	2013–2020	2003–2012	2013–2020	1993–2020
MSLA	18.8	30.5	38.7	100	32	169
TSLA	− 19	− 16.3	13.6	14	183	172
HSLA	0.6	0.81	0.43	35	− 47	− 28
SSLA	− 18.6	− 15.5	14.1	17	191	175
Precipitation	0.15	0.13	0.44	− 13	238	193
Evaporation	− 0.17	− 0.06	− 0.15	65	150	12
Runoff	− 0.15	− 0.09	0.21	40	333	240
AtmPressure	4.9	− 16.5	− 5.5	− 437	67	− 212
WSC	0.004	0.002	− 0.004	− 50	− 300	− 200
Current	0.2	0.12	− 0.07	− 40	− 158	− 185
NHF	− 7.2	− 1.007	0.08	86	108	101
OHC	− 8.6e8	− 7.5e8	6.6e8	13	188	176

### Leading modes of interannual MSLA variability

The following section discusses the results of the EOF analysis to further investigate the climate and oceanic phenomena that dominate the interannual variability of MSLA in the GoG. This investigation was conducted using the detrended spatial MSLA dataset, which isolates interannual variability by removing long-term effects from the time series data.

**Figure 4:**
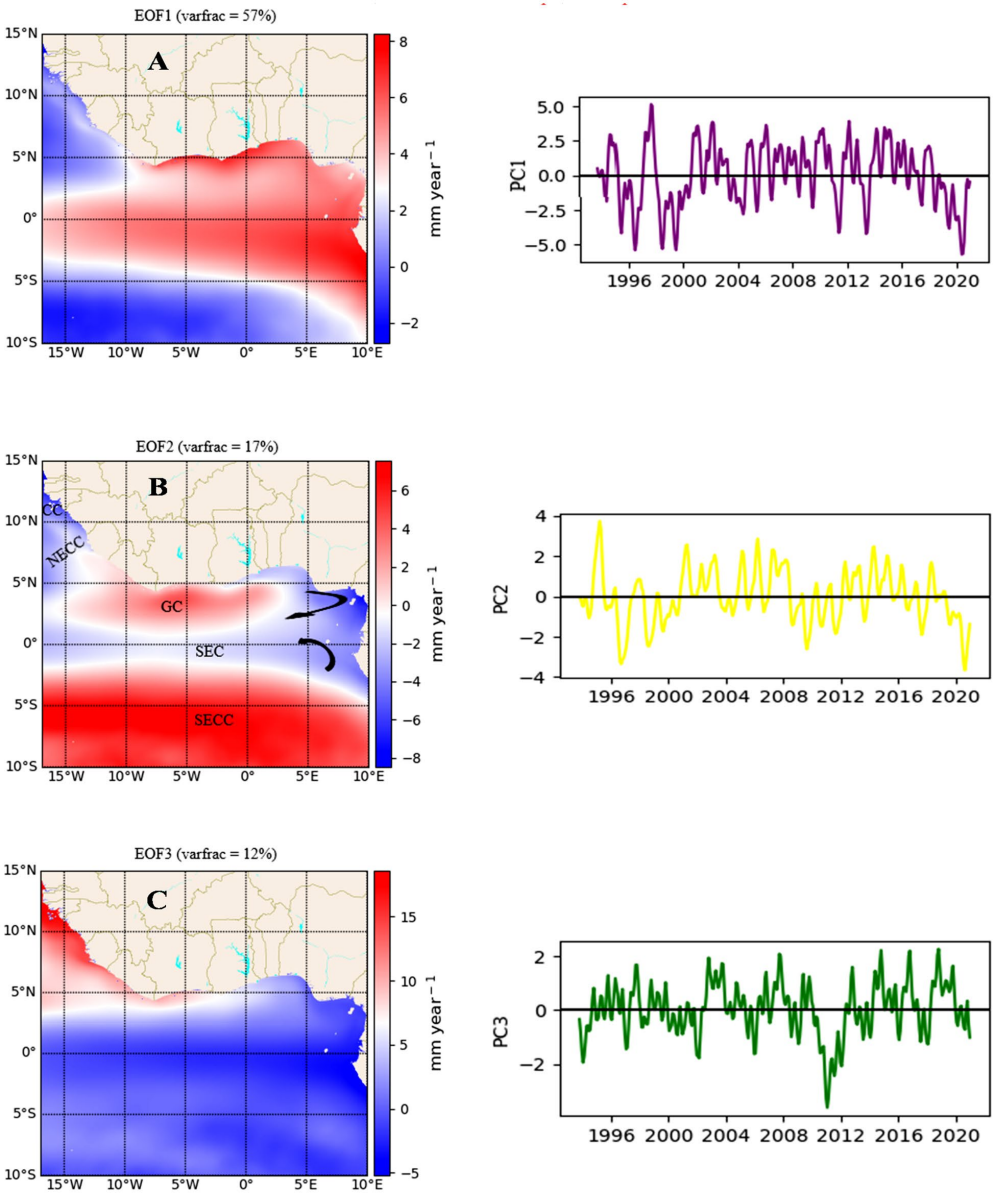
The first three (**A**–**C**) leading EOF modes of the filtered and detrended MSLA estimated over the period 1993–2020, along with their time series Principal Components (PCs) on the right. The labeling in B showcases the five principal currents and their flow regions, forming the circulation pattern in the Gulf of Guinea: SECC (south equatorial counter-current), SEC (south equatorial current), GC (Guinea current), NECC (north equatorial counter-current), and CC (canary current). The calculations and the maps were produced with a Python (3.10.9) script.

The first mode, EOF1, which is the dominant mode (Fig. 4A), explains 57% of the total variance in MSLA. This mode exhibits a mountainous tripole pattern. The base of the mountain displays high variability of MSLA, culminating from the eastern coasts and extending up to the coasts of Côte d'Ivoire and parts of Liberia in the west. It has a shoulder extending to the equatorial region, with the slopes of the mountain displaying moderately high sea levels that decay with increasing latitudes on both sides of the slope (north and south basins). These features demonstrate the classical characteristic of the barotropic Kelvin waves (equatorial and boundary) as described by^25^. “The wave propagates equatorward along the western boundary, poleward along the eastern boundary, and cyclonic circulation around a closed boundary (counterclockwise in the Northern Hemisphere and clockwise in the Southern Hemisphere). It has the highest amplitude at the boundary which exponentially decays away from the boundary. Furthermore, it consistently propagates eastward at the equator, attaining its maximum magnitude and subsequently decaying exponentially with increasing latitude”. This wave is usually wind-induced due to atmospheric pressure gradients acting on the ocean surface. This suggests that the teleconnectivity of the equatorial and eastward propagation of Kelvin waves modulate the interannual sea level in the GoG, with the highest contribution observed around 1998 and a persistent slowdown during the 2016–2020 periods.

The second mode, EOF2, accounts for 17% of the variance fraction and exhibits a tripole pattern. It shows low sea levels along the northwest and northeast coasts, surrounding a high sea level in the central north coasts. The low sea level extends to the equatorial basin, separating the two high sea levels in the central north coasts and the south basin. This pattern closely corresponds to the temperature distribution of the surface oceanic circulation in the GoG, primarily driven by wind (Fig. 4B).

The third EOF of the interannual variability of MSLA explains 12% of the total variance and exhibits a meridional dipole pattern with high variability observed in the northern basin, a pattern that bears the hallmark of Atlantic Meridional Overturning Circulation (AMOC). AMOC is a powerful oceanic current system that plays a crucial role in regulating the climate in the Atlantic by transporting warm surface water northward and cold deep water southward^26^. The temperature distribution by AMOC consequently affects the sea level variability as observed in EOF3 (Fig. 4C).

### Seasonal variability

**Figure 5:**
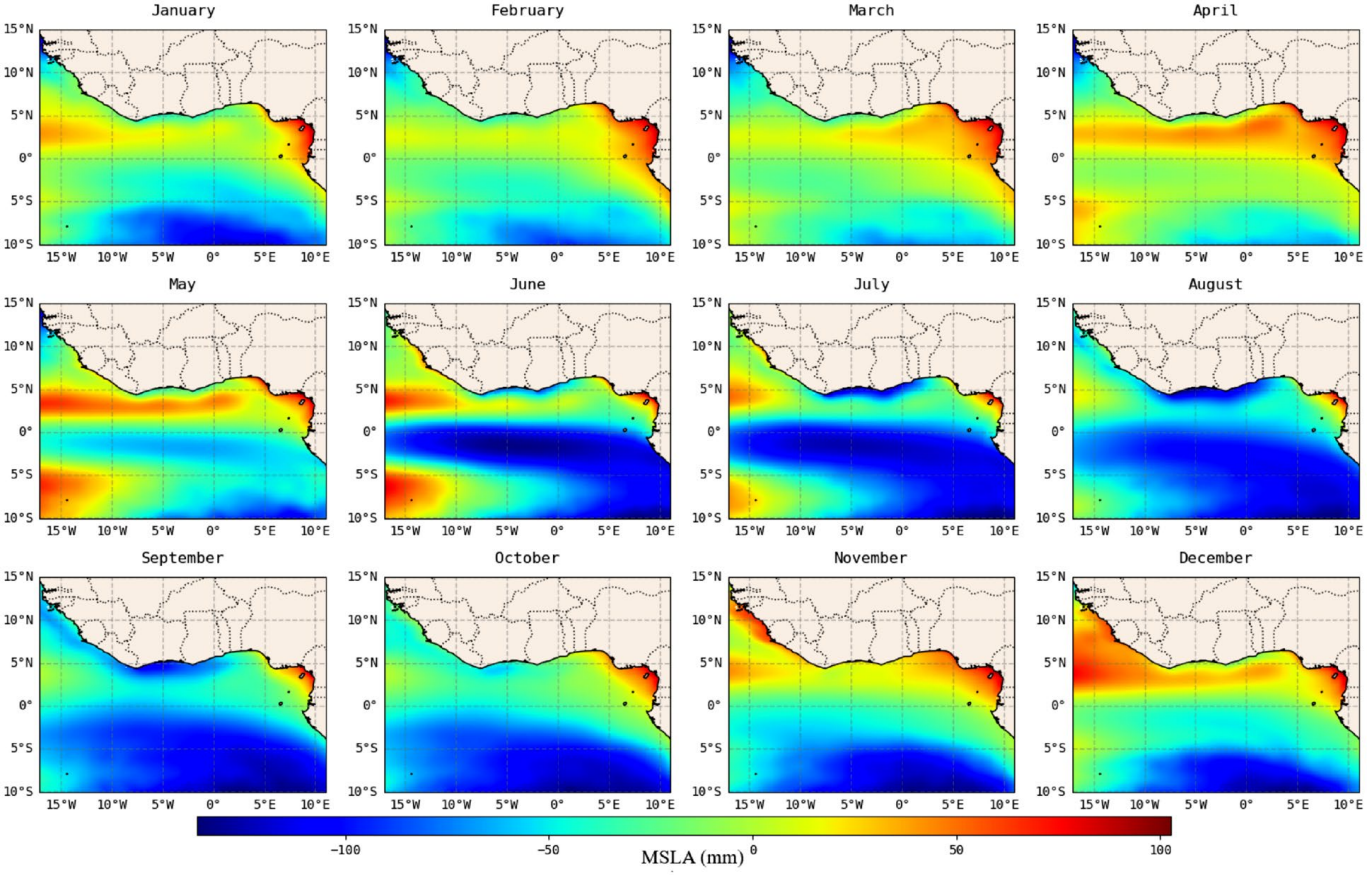
Spatial trend of seasonal variability of MSLA obtained by calculating the mean value of undetrended MSLA for each month at each grid point for the periods 1993–2020 in the Gulf of Guinea. The calculations and the maps were produced with a Python (3.10.9) script.

Analysis of the spatial seasonal variability of MSLA in the GoG, as depicted in Fig. 5, shows a distinct spatial seasonal variability of sea level across the basin. The northern basin, which has the highest MSLA distribution throughout the seasons compared to the southern basin, records its lowest value towards the end of RONS, through the ROFFS seasons. Notably, Cameroon and Nigerian coasts have an overall highest seasonal sea level trend compared to other coasts with the highest during RONS season. This is because the highest sea level along the northwest coasts is observed in December (DMON). This seasonal sea level pattern in the northern basin is similar to that of the south, with the highest in RONS, specifically in the southwestern basin.

**Figure 6:**
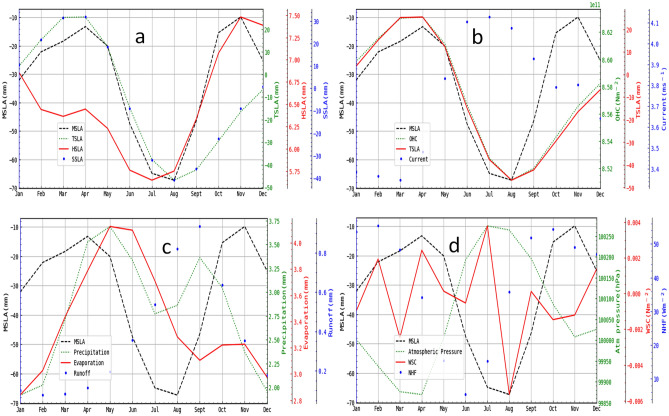
Seasonal trend and variability of MSLA and its forcings obtained by calculating the mean value of the basin average of undetrended MSLA and its forcings for each month. The forcings are partitioned according to their potential source components: (**a**) steric component, (**b**) oceanic component, (**c**) hydrological component, and (**d**) atmospheric component.

The overall seasonal trend of MSLA across the basin (Fig. 6) shows two distinct peaks (April and November) and troughs (July and August), with November and August being the highest and lowest troughs, respectively. These peaks and troughs correspond to the observed HSLA peaks and TSLA troughs (Fig. 6a). Noticeably, the first peak of MSLA was preceded by the current trough in March, and the current peak in July preceded the MSLA trough (Fig. 6b). However, OHC, which follows a similar pattern with SSLA, shows a striking resemblance to the seasonal pattern of MSLA, with only a deviation in the period of the observed highest MSLA peak. Meanwhile, the atmospheric pressure demonstrates an inverse relationship with MSLA, rising as the sea level falls and vice versa throughout the season (Fig. 6d). WSC exhibits a seasonal pattern similar to the ocean current as they lead MSLA by a month. The same is observed for NHF; however, the impact of NHF on MSLA contrasts with both current and WSC, as MSLA peaks precede NHF peaks.

### Correlations between MSLA and the forcing variables

**Figure 7:**
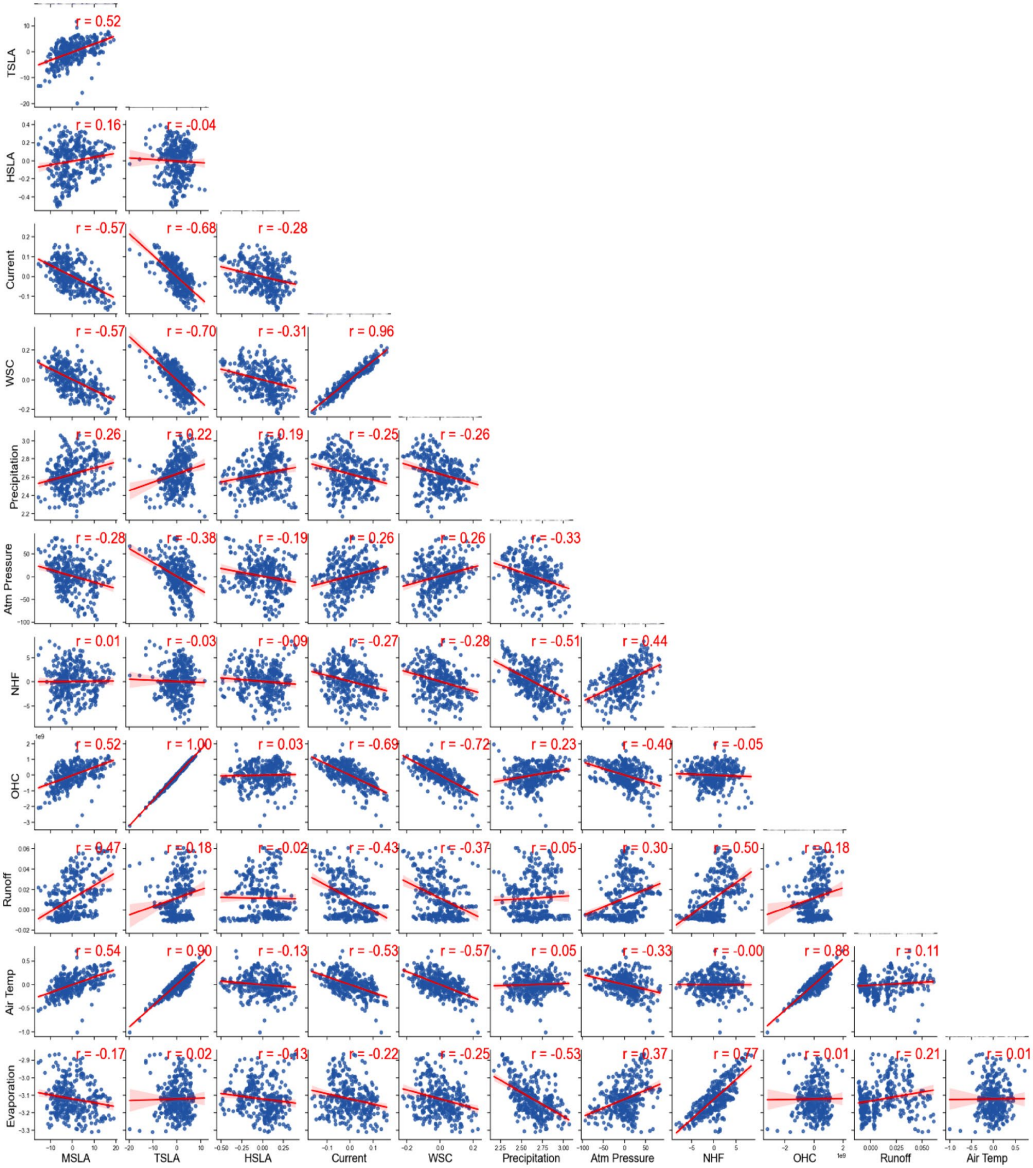
Correlations (values at the top of each subplot) and regression fits (red solid lines) among the detrended and filtered mean sea level (MSLA) and its forcings, including steric sea level anomaly (SSLA), thermosteric sea level anomaly (TSLA), halosteric sea level anomaly (HSLA), ocean heat content (OHC), wind stress curl (WSC), air temperature, net heat flux (NHF), precipitation, evaporation, freshwater runoff, and atmospheric pressure for the period 1993 to 2002 in the Gulf of Guinea.

The results of the Pearson correlation and regression analysis conducted on the detrended and filtered MSLA and its forcings in GoG are presented in Fig. 7. Our analysis revealed a significant level of association between MSLA and its various forcing variables. This connection provides a comprehensive understanding of the intricate relationships at play within the GoG. For instance, we observed that MSLA exhibited a positive relationship with SSLA, TSLA, HSLA, OHC, NHF, precipitation, freshwater runoff, and air temperature. These positive associations highlight the interdependence of these variables, emphasizing how changes in one component can influence MSLA and, in turn, contribute to the sea level variability in the GoG. Conversely, we noted negative relationships between MSLA and certain other variables, including current, WSC, atmospheric pressure, and evaporation. These negative associations provide additional depth to our understanding of the complex interactions governing sea level changes. They reveal the counteracting forces at play, where these variables act in opposition to MSLA, influencing sea levels by exerting forces in different directions. Additional observations include strong positive correlations between current and WSC, evaporation and NHF, air temperature with OHC and TSLA, and vice versa. On the other hand, strong negative correlations are found between precipitation and evaporation, TSLA and OHC with current and WSC, and vice versa. These findings further elucidate the intricate relationships between MSLA and its forcing variables, shedding light on the complex dynamics of sea level variability in the GoG.

### Model performance

**Figure 8:**
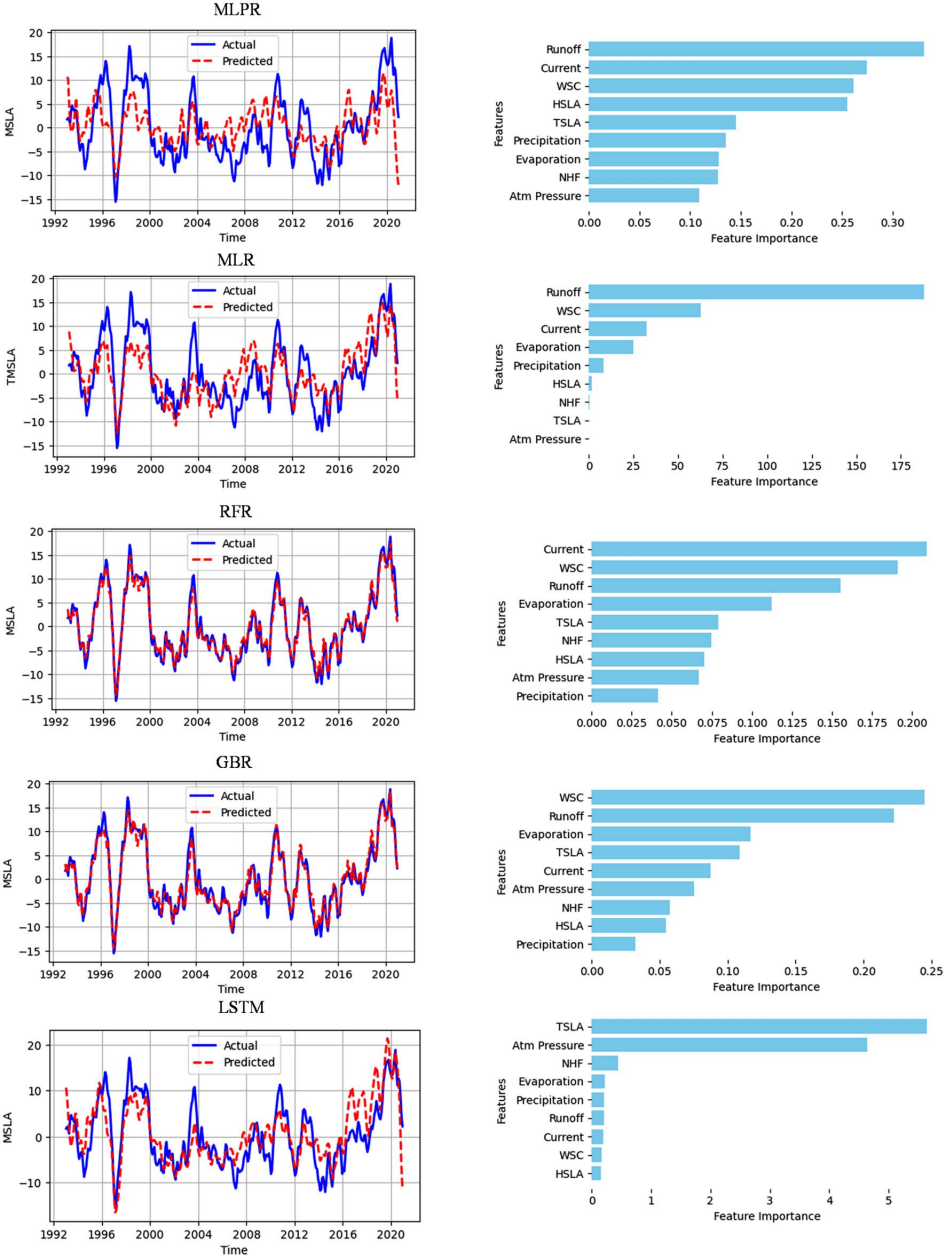
Comparison of the predicted and actual values of MSLA predicted by each of the models (i.e. MLPR, MLR, RFR, GBF, and LSTM) over the period 1993–2020, along with their feature importance on the right in the Gulf of Guinea.

Following the model procedures and evaluation metrics presented in the methodology section of this paper, we present the results of the model performance and their associated feature importance in Fig. 8. Interestingly, we found no significant difference in the models' performance between splitting the data and using the entire dataset for training and testing. Therefore, we depict the plots of the model where we use the entire dataset for training and testing to show the full temporal extent of the data. However, in practical terms, such as for model deployment, the splitting model is considered standard. Our observations show that RFR and GBM models with ${R}^{2}$ and RMSE of 0.97, 0.96, and 1.14, 1.36 respectively, exhibit the best performance among the evaluated models in this study. However, the relative importance of input features (predictor variables) in making predictions or explaining the target variable's variance varies among the models. While it was demonstrated that current and WSC are related in the previous section of this paper in the GoG, they play dominant roles in the performance of RFR and GBM models, respectively. Additionally, the LSTM model, outperforming MLR and MLPR models, closely follows RFR and GBM, with TSLA emerging as the most influential variable. Notably, freshwater runoff stands out by dominating over other features in both MLR and MLPR models. However, the MLPR model exhibits the lowest performance when compared to all the models considered in the study. In general, the performance of machine learning models depends on several factors, including the complexity of the model, data distribution, feature selection methods, hyperparameter tuning, and the model's assumptions about the datasets. The summary of the results of the models’ evaluation metrics is presented in Table 2.

**Table 2:** Summary of the result of model evaluations, including MLPR, MLR, FRF, GBR, and LSTM.

Model	Evaluation metrics	
	${R}^{2}$	RMSE
MLPR	0.50	5.41
MLR	0.72	4.48
RFR	0.97	1.14
GBR	0.96	1.36
LSTM	0.75	4.46

## Discussion/conclusion

In this study, we have undertaken a comprehensive analysis of the linear trend of MSLA, focusing on the changes in the decadal trend and their underlying drivers in the GoG. Our investigation involved separate examinations of decadal trends for the periods 1993–2002, 2003–2012, and 2013–2020. Additionally, we explored the seasonal variability of MSLA and investigated potential links between interannual sea level variability and large-scale oceanic and atmospheric forcings spanning from 1993 to 2020. To model sea levels in the GoG, we assessed various supervised machine learning models, including artificial neural networks such as LSTM and MLPR, alongside traditional regression methods like MLR, GBM, and RFR.

Our analysis revealed a consistent increase in the linear trend of MSLA across the entire basin, with the northern region exhibiting a more pronounced trend. The total linear trend from 1993 to 2020 amounts to approximately $88\, \mathrm{mm}$. The highest decadal trend ($38.7\, \mathrm{mm}$) was observed during 2013–2020, while the most substantial percentage increment occurred during 2003–2012 (100%). Zonal differences were evident in the variability of the linear trend of sea level across decades, with the western region showing unique behavior during the 2013–2020 period. The spatial variabilities of the linear decadal sea level trend across the basin are driven by variations in physical forcings within the sub-basins. These forcings, SSLA, TSLA, HSLA, OHC, WSC, air temperature, atmospheric pressure, NHF, precipitation, evaporation, and freshwater runoff, exert distinct impacts on sea levels. While WSC, current velocity, atmospheric pressure, and evaporation negatively correlate with sea levels, SSLA, TSLA, HSLA, OHC, air temperature, NHF, precipitation, and freshwater runoff exhibit positive associations.

Our study also highlighted the significant influence of large-scale oceanic and atmospheric phenomena on the spatial distribution of sea levels. The first three modes of Empirical Orthogonal Function (EOF) variability explained substantial proportions of variance, with the first mode reflecting teleconnections between equatorial and coastal Kelvin waves driven by atmospheric circulations. However, the role of Kelvin waves in modulating the interannual sea level variability near the coast is well-documented^27,28^.

The second mode revealed the spatial variability of the current circulation system and its thermal characteristics, largely governed by the wind system. For instance, in the northern basin, the relatively cooled Canary Current (CC) flows southward along the African coasts between 30° and 10°N^29^. Meanwhile, the warm North Equatorial Counter Current (NECC) flows between 3° and 10°N, acting as the northern limit for the South Equatorial Current (SEC), and the Guinea Current (GC) is a relatively warm eastern flowing current between 3°-5°N along the coasts of West Africa^30^. In the southern basin, the SEC flows westward between 2°N and 4°S and is fed with relatively cool Benguela water, while the South Equatorial Counter Current (SECC) is a relatively warm eastward flowing current that moves below the SEC^31^. This confirms the temperature distribution of the surface circulation as the second leading mode of interannual variability of MSLA in the GoG. The highest variability was observed in 1994 and has consistently decreased during the 2017–2020 period.

The third mode captured the AMOC, a vital oceanic process controlled by density-driven currents. While there have been reports indicating a slowdown of AMOC in recent decades^32^, more recent research suggests that AMOC may already be recovering^33,34^. For instance, the results of the analysis of the interannual variability of AMOC conducted between 2004 and 2018 by^33^ along 26°N, which is slightly above our study area, show a significant decline between the period 2009–2010 and two peaks between the periods 2013–2014 and 2018. This result is consistent with the interannual variability of MSLA in the GoG, as observed in PC3. While the variability in the AMOC has been linked to the sea level variability in the GoG^35^, to the best of our knowledge, no research has reported the interannual variability of AMOC in the GoG. Therefore, the work of Moat et al. could offer valuable comparative insights, as most of the variability in AMOC originates from the tropical Atlantic.

Furthermore, seasonal variability in sea level trends emerged due to seasonal changes in forcing factors. The opposing impact of current and WSC and the positive effect of NHF led MSLA by a month, just as other variables experience seasonal fluctuations with MSLA.

Despite the challenges inherent in sea level modeling and prediction, the integration of advanced artificial neural networks and machine learning techniques presents a promising solution. By harnessing extensive datasets encompassing ocean currents, WSC, freshwater runoff, TSLA, HSLA, NHF, and atmospheric pressure, these innovative approaches can unveil hidden relationships and underlying mechanisms shaping oceanic processes and sea levels. Such integration empowers the development of more realistic models, expanding projection capabilities over extended temporal ranges. Notably, our analysis highlighted the efficacy of RFR and GBM models, with accuracy rates of 97% and 96%, respectively, in reproducing interannual sea level patterns in the Gulf of Guinea. The implications of the findings of this work may be extended to other regions in terms of methodological transferability for regional sea level modeling, understanding the historical trend and their drivers, enabling proper environmental monitoring, climate adaptation, resilience, and data-driven decision-making.

## Methodology

### Study area

**Figure 9:**
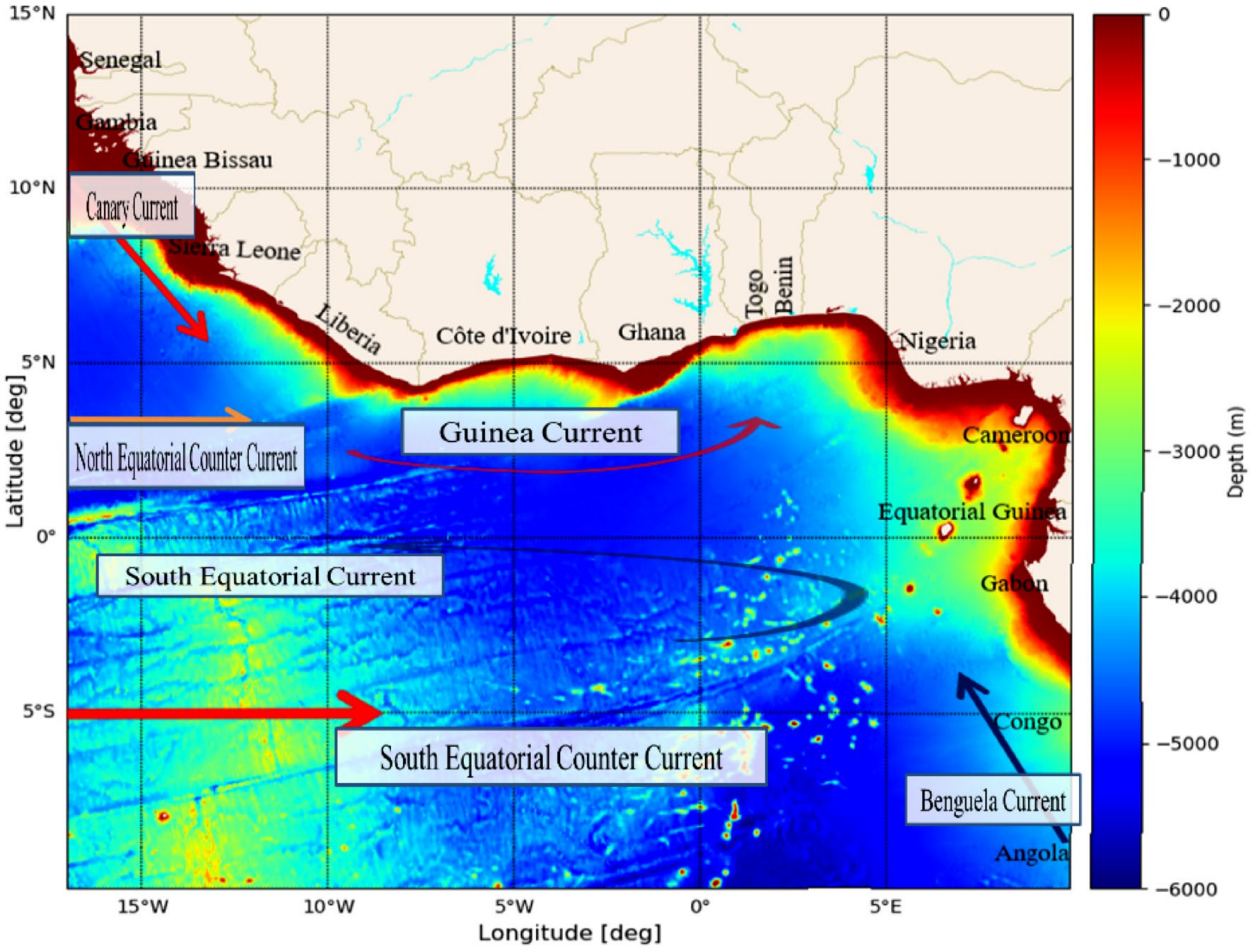
The study area map, featuring countries, principal currents, and bathymetry in the Gulf of Guinea. The bathymetry data is based on a global 15-arc-second interval grid obtained from GEBCO (https://www.gebco.net) with a minimum depth of 10 m. The map was generated using a Python (3.10.9) software. The Python script incorporated geographical data and employed libraries such as Matplotlib and Geopandas to create the map.

The Gulf of Guinea, situated along the western coast of Africa, stretches from Cape López near the equator to Cape Palmas, spanning longitudes ${17}^{o}W$ to ${11}^{o}E$, and latitude ${15}^{o}N$ to ${10}^{o}S$ (Fig. 9). Known for its predominantly low-lying coasts, the Gulf features warm tropical waters with relatively low salinity due to the influx of major rivers, including the Volta, Niger, Congo, Forcadoes, Ouémé, Delta, Sassandra, Tano, Nun, and Komoé, among others. Spanning approximately 6000 km, the Gulf boasts a diverse coastline, characterized by a nearly uniformly narrow continental shelf measuring about 100 nautical miles. The region is impacted by five principal ocean currents: the Benguela Current, Canary Current, South Equatorial Current, Counter Equatorial Current, and Guinea Current^36^. The prevailing climate follows a monsoon pattern, particularly the West Africa monsoon (WAM), with two primary air masses: the Southwest and Northeast winds^37^. The region experiences minimal dry season during the summer, leading to two distinct wet seasons annually^38^, marked by the onset and conclusion of the rainy period. The initiation of the rainy season is often accompanied by low rainfall amounts, referred to as pre-rain onset^39^. This study classifies seasons based on West Africa's continental rainfall quantity and timing. The winter season (DJF) is identified as the dry-monsoon (DMON), spring (MAM) as pre-rain onset (PRONS), summer (JJA) as rain onset (RONS), and autumn (SON) as the rain-offset season (ROFFS).

### Data source

The temperature and salinity data utilized in this research are drawn from the high-resolution 3-D GLORYS12V1 products, with a spatial resolution of 1/12 degrees (~ 9 km), spanning the period from 1993 to 2020. These products originate from the Nucleus for European Modeling of the Ocean (NEMO) general circulation model^40^, incorporating surface boundary conditions from the European Centre for Medium-Range Weather Forecasts (ECMWF) atmospheric reanalysis and forecasts. Through the assimilation of near-real-time observations, the NEMO model offers accurate estimates of the oceanic state in the GoG. This dataset has undergone rigorous validation against in situ observations and other sea surface temperature (SST) and salinity products, demonstrating robust consistency with independent data sources (e.g.,^41–43^). Its applicability spans oceanographic and climate research domains (e.g.,^44–47^). Accessible from the Copernicus Marine Environment Monitoring Services CMEMS data archive, the dataset covers the temporal span from 1993 to the present.

For sea level data, the monthly gridded sea surface height, hereafter referred to as MSLA, was acquired for the GoG from CMEMS. This dataset amalgamates observations from diverse altimetry missions, resulting in a consistent and unbiased dataset characterized by a 1/4° horizontal and vertical spatial resolution^48^. Necessary geophysical corrections, including tidal corrections using the Finite Element Solution 2014 (FES2014) ocean tide model^49^, were performed by the Data Unification and Altimeter Combination System (DUACS) to produce the dataset. Additionally, the dataset underwent further refinement for glacial isostatic adjustment (GIA) using the ICE5G-VM2 GIA model^50^ to isolate oceanographic phenomena. Widely employed by researchers in investigating sea level variability, ocean dynamics, and coastal processes (e.g.,^24,51,52^), the data was accessed from the CMEMS archive at http://marine.copernicus.eu/. Furthermore, supplementary datasets encompassing air temperature, u wind (10 m), v wind (10 m), total precipitation, evaporation, Atmospheric pressure, net shortwave radiation, net longwave radiation, surface latent heat flux, and sensible heat flux at a single pressure level were sourced from the European Centre for Medium-range Weather Forecasts^53^ reanalysis era5 data, accessible at http://cds.climate.copernicus.eu/.

### Parameterization

TSLA and HSLA were computed using the Thermodynamic Equation Of Seawater 2010 (TEOS-10), which comprises a set of standardized equations for determining the thermodynamic properties of seawater. TSLA and HSLA account for the individual impact of temperature and salinity, respectively, which can cause expansion or contraction of sea level depending on their respective values at a specific location and time. Their combined impact forms the steric sea level anomaly (SSLA), which measures how changes in water density affect the sea level. The steric sea level from the surface up to 1000 m is computed following^54,55^):

1 $$h={h}_{T}+ {h}_{S} = {\int }_{-1000}^{0}\alpha \left(T- {T}_{o}\right)dz+{\int }_{-1000}^{0}\beta \left(S- {S}_{o}\right)dz$$

where h is the total steric sea level height, ${h}_{T}$ and ${h}_{S}$ are the thermohaline and halosteric components, respectively. T and S represent the temperature and salinity at each grid point, while ${T}_{o}$ and ${S}_{o}$ denote the reference temperature and salinity. α and β are the thermal expansion and haline contraction coefficients, respectively, calculated from the temperature and salinity using the (TEOS-10) equation.

Similarly, wind speed and wind stress were calculated from ERA5 zonal (u) and meridional (v) wind components data using the following equations:

2 $$G= {({u}^{2}+ {v}^{2})}^{0.5}$$

3 $$K={({\tau }_{x}+ {\tau }_{y})}^{0.5}$$

here u and v represent the u and v components of wind, G is the wind speed, K is the wind stress, and ${\tau }_{x}$ and ${\tau }_{y}$ are defined as ${\rho }_{air}cd \times G\times u$ and ${\rho }_{air}cd \times G\times v$, respectively, which are the wind stress of the u and v components. ${\rho }_{air}$ represents air density, and cd is the drag coefficient. The curl of the wind stress is computed as follows:

4 $${curl}_{z }K= \frac{{\partial \tau }_{y}}{\partial x}- \frac{{\partial \tau }_{x}}{\partial y}$$

where ${\tau }_{x}$ is the zonal wind stress component, ${\tau }_{y}$ is the meridional wind stress component, and x and y represent eastward and northward coordinates, respectively. Also, the ocean heat content (OHC) within 1000 m along the GoG is calculated following^56^:

5 $$OHC= \rho {C}_{p}{\int }_{-1000}^{0}\left[T(z)\right]dz$$

where ρ is the seawater density calculated from temperature and salinity at each grid point following^57^, ${C}_{p}$ is the specific heat capacity of seawater (4178 J kg^−1^ °C^−1^), and T(z) is the temperature (℃) at each grid point. Finally, the surface net heat flux (NHF) is estimated following^58^:

6 $${\text{NHF}} = {\text{SWR}} + {\text{LWR}} + {\text{LHF}} + {\text{SHF}}$$

where the respective components of Eq. 6 are defined as follows: net shortwave radiation (SWR), net long-wave radiation (LWR), surface latent heat flux (LHF), and sensible heat flux (SHF).

### Procedure

The spatial decadal linear trends of MSLA are calculated at each grid point by taking a ten-year average of the annual trends in the study domain using regression coefficients estimated by the ordinary least squares method. The significance of these trends is tested using a non-parametric Mann–Kendall (MK) trend test with a 99% confidence level^59,60^. Thereafter, a non-parametric Theil-Sen’s slope estimator^61,62^ was employed to estimate the magnitude and direction of trends in the time-series data. To isolate the interannual variability from the datasets, monthly mean values were extracted across the entire longitude and latitude. The climatological mean was then removed from the extracted time series data before they were detrended and filtered using a low-pass filter with a 13-month running mean. This approach considers that detrending isolates interannual variation in climate variables^63^. A similar approach was employed to isolate the interannual spatial variability from the MSLA at each grid point. Subsequently, Empirical Orthogonal Function (EOF) analysis was performed. EOF analyses are often used for dimensionality reduction and the extraction of dominant spatial patterns of climate variability and how they change with time^64^. This mathematical technique decomposes datasets into a set of orthogonal patterns (EOFs) and their corresponding time series Principal Components (PCs). Each EOF represents a spatial pattern of variability, and the PCs indicate how these patterns vary over time. In our study, EOF analysis was employed to gain insights into the spatial patterns and temporal variations of interannual MSLA and its connection with large climate and oceanic phenomena. Meanwhile, the spatial seasonal signal was extracted from the undetrended MSLA by calculating the mean value of sea level for each month at each grid point. Additionally, the linear seasonal signal was extracted from the undetrended MSLA and its forcings by calculating the mean value of the basin-average sea level for each month. Five different ANN and MLT models, namely MLPR, MLR, RFR, GBR, and LSTM, were developed to determine the best-performing model for sea level predictions in the GoG. The description of each model is provided below.

### Model description

#### Multi-layer perceptron regression (MLPR)

**Figure 10:**
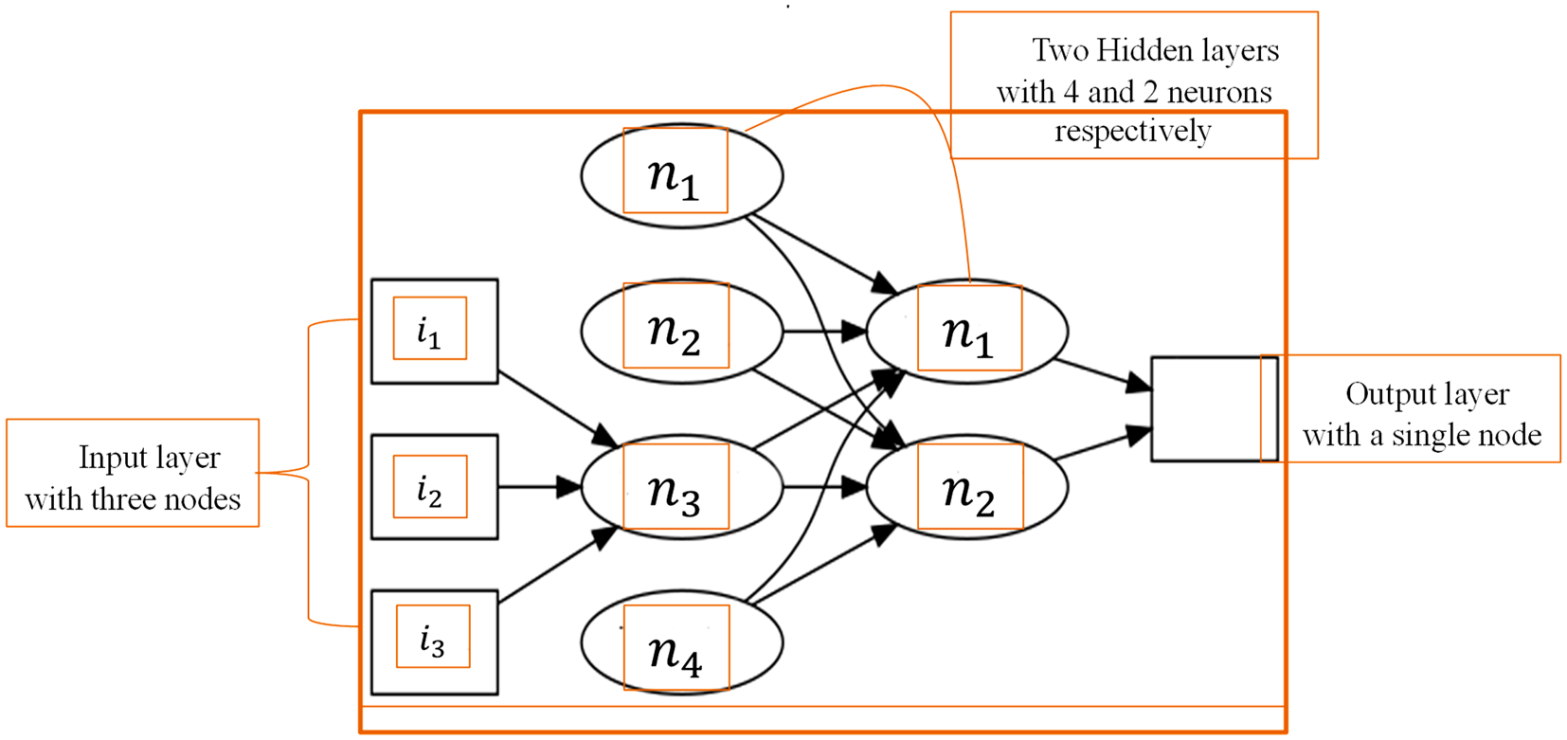
Architecture of a Multi-Layer Perceptron (MLP) with Two Hidden Layers. The input layer consists of three nodes, representing the three input features. The network contains two hidden layers with 4 and 2 neurons, respectively. Each neuron in the hidden layers applies an activation function to the weighted sum of its inputs to introduce non-linearity and enable learning complex patterns. Finally, the output layer has a single node, which generates the predicted continuous value for regression tasks. The architecture of this MLP allows for the processing and transformation of input data through the feedforward process, leading to accurate predictions based on the provided inputs.

MLPR is an ANN model specifically designed for regression tasks, It utilizes an FNN network model to predict continuous numerical values rather than discrete categories. The network comprises multiple hidden layers of neurons that apply nonlinear transformations to the input data, enabling the model to learn intricate patterns and relationships between input features and the target output. Its effectiveness in sea level prediction has been well-documented in previous studies^21,65^. The MLPRegressor model architecture, depicted in Fig. 10, illustrates the key components, including the input layer, hidden layers, and output layer. Neurons are the fundamental building blocks of ANN that process information and facilitate the network's ability to learn complex patterns and make predictions based on input data. Each neuron applies an activation function, such as the rectified linear unit (ReLU), to the weighted sum of its inputs. This helps address the vanishing and exploding gradient problem^66^ and is defined by the function in Eq. (7)

7 $$\mathrm{ReLU}=\mathrm{ max}\left(0,\mathrm{ x}\right)$$

where x represents the weighted sum of inputs to the neuron. The weighted sum of inputs to a neuron (Z), which captures relevant features and introduces non-linearity, is calculated by multiplying the input values with their corresponding weights and summing them up, along with the bias term, as represented by Eq. (8).

8 $$Z = (W_{i} .X_{i} ) + b$$

where ${W}_{i}$ represents the weights, ${X}_{i}$ represents the input values, and 'b' is the bias term. Feedforward is a fundamental concept in neural networks that defines the process of propagating input data to the output through the network's layers without any feedback connections. This sequential flow of information in a single direction allows the network to make predictions based solely on the provided input data. The feedforward process is aided by a series of layers of neurons, with the activation of each neuron determined by Eq. (9):

9 $${A}_{i}= \mathrm{ReLU}\left({Z}_{i}\right)$$

where ${A}_{i}$ represents the activation of neuron i, and ${Z}_{i}$ represents the weighted sum of inputs to neuron i. The output of the model is calculated based on the activations of the neurons in the output layer i.e. the weighted sum of activations without applying any additional activation function and it expresses as:

10 $$Y= \sum \left({W}_{i} . {A}_{i}\right)$$

where Y represents the predicted output, ${W}_{i}$ represents the weights connecting the neurons, and ${A}_{i}$ represents the activations of the neurons in the output layer.

#### Multiple linear regression (MLR)

MLR is a statistical technique that uses a linear equation to establish the relationship between multiple independent variables (predictors) and a dependent variable (response). It determines the best-fitting line between the dependent variable and the independent variables by employing a least squares technique to estimate the independent variables for predicting the dependent variable, assuming a linear relationship between the variables. The application of MLR in modeling and prediction tasks in the fields of oceanography and climate science has been extensively documented^67,68^. However, MLR has some limitations, such as the linearity assumption, independence assumption, multicollinearity, sensitivity to outliers, and limited handling of non-normality^69–71^. The MLR model equation can be represented as follows:

11 $$Y = C + \beta_{1} x_{1} + \beta_{2} x_{2} + \beta_{3} x_{3} + \ldots \beta_{n} x_{n} + \varepsilon$$

where Y represents the dependent variable (response variable), C represents the intercept (constant term), ${\beta }_{1}$, ${\beta }_{2}$, …, ${\beta }_{n}$ represent the regression coefficients associated with the independent variables ${x}_{1}$, ${x}_{2}$, …, ${x}_{n}$ and ɛ represents the error term, accounting for unexplained variation.

#### Random forrest regression (RFR)

**Figure 11:**
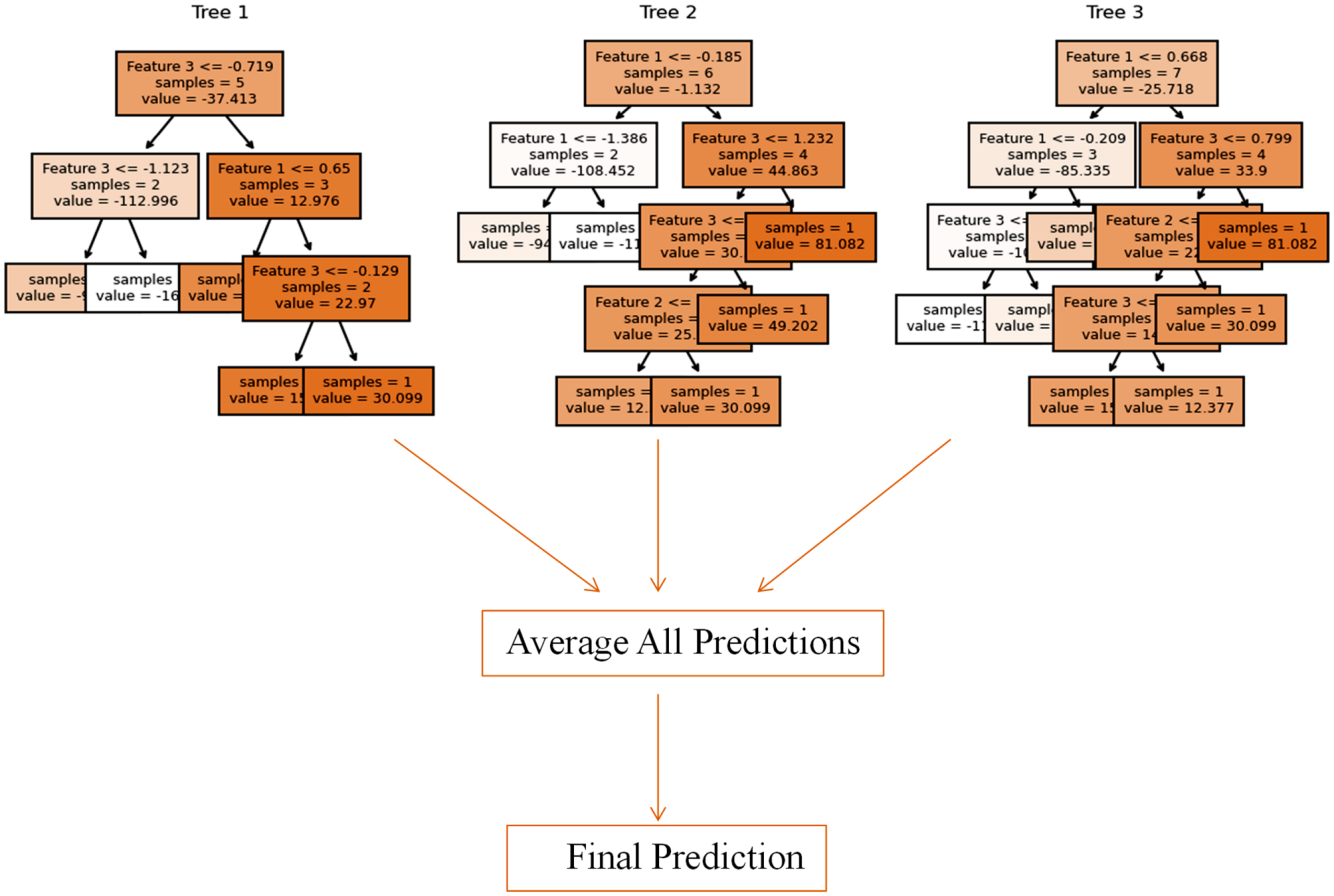
The architecture of the Random Forest Regression (RFR) Model. The architecture has three decision trees as estimators in the random forest ensemble. Each decision tree operates independently, with the depth varying based on the data and problem complexity. The input data contains three features, which are used to train and construct the three decision trees. The RFR model effectively aggregates the predictions of these decision trees to make robust and accurate regression predictions.

RFR is an ensemble machine learning technique that addresses the limitations of individual decision trees by combining them through bagging. This results in a robust and accurate regression model. Figure 11 illustrates the architecture of RFR with multiple decision trees. The structural arrangement of the trees provides insights into the internal workings of the RFR model, enabling a better understanding and interpretation of its predictions. Unlike other machine learning models, each RFR decision tree in the ensemble operates independently and does not have explicit equations associated with its components. However, the RFR architecture involves three key steps: bootstrap sampling, feature subset selection, and the final prediction. Bootstrap sampling is used to create subsets of training data. Then, feature subset selection is performed to reduce the correlation between the trees, and the final prediction is obtained by aggregating the individual predictions of the decision trees, usually by taking the mean or median. This approach introduces randomness through bootstrapping and feature subset selection, effectively reducing overfitting and improving robustness. RFR is highly effective in handling noise, capturing complex relationships, and generating accurate predictions by leveraging the collective power of multiple trees^72^. Furthermore, the application of RFR for sea level prediction has been extensively documented in the literature^73–75^.

#### Gradient boosting machine (GBM)

**Figure 12:**
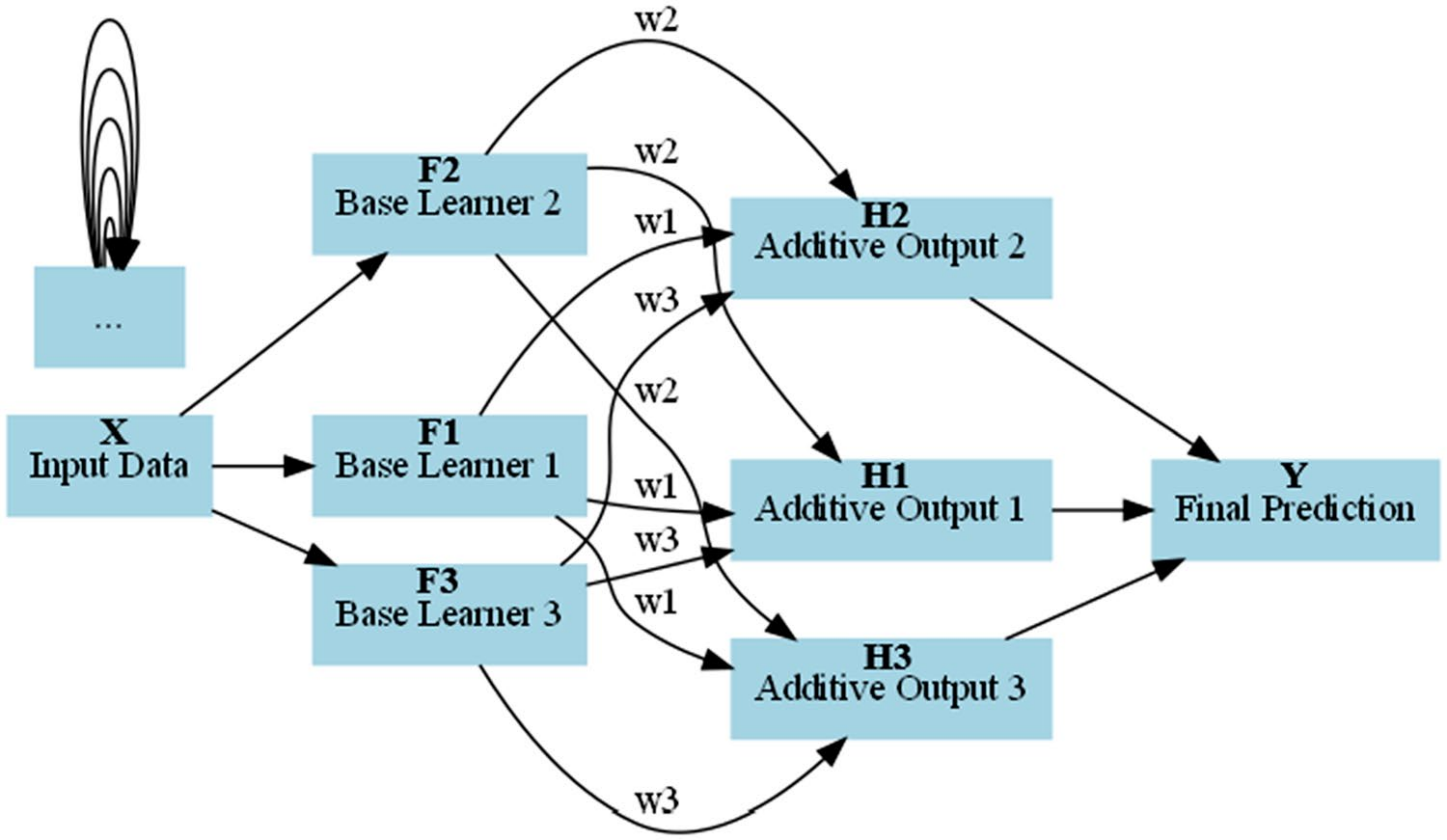
The architecture of the Gradient Boosting model. It consists of multiple Base Learners (F1, F2, F3) and Additive Outputs (H1, H2, H3), which collectively contribute to the final prediction (Y). The input data (X) represents the input features, and the number of features can be determined by the dimensionality of the input data. The number of Base Learners is determined by the number of estimators specified when creating the model. The 'w1', 'w2', and 'w3', represent the contribution weights of each Base Learner to the final prediction. The final prediction (Y) is the output of the Gradient Boosting model, which is a combination of the additive outputs from all Base Learners.

GBM, a machine learning algorithm leveraging an ensemble method called boosting, has gained significant popularity for its effectiveness in regression tasks, handling complex non-linear relationships and interactions between variables. Introduced by^76^ and further elaborated in 2001, GBM generates a robust predictive model by combining multiple weaker models. The fundamental principle involves iteratively training a sequence of models, typically decision trees, to refine overall prediction accuracy by rectifying errors made by previous models. This iterative process ensures subsequent models focus on areas where previous ones underperformed, resulting in a powerful and accurate predictive model. GBM demonstrates robustness in handling noisy data, outliers, and missing values. Figure 12 illustrates the GBM model architecture, showcasing core concepts of gradient boosting and its sequential nature. It integrates multiple base learners to form a strong ensemble prediction, depicting the underlying mechanism. The GBM architecture consists of input data, multiple base learners capturing different patterns and relationships, additive outputs from each base learner, and the final prediction from the combined additive outputs. Weighted edges (w1, w2, and w3) connect nodes, determining each base learner's contribution to the additive outputs and the ultimate prediction. The base learner represented as a function ${F}_{i}\left(x\right)$, is denoted by index i and input data x. Thus, the base learner equation is expressed as:

12 $${F}_{i}\left(x\right)= {f}_{i}\left(x\right)$$

Here, ${f}_{i}\left(x\right)$ represents the prediction of the i-th base learner. The equation for the additive output of the i-th base learner (${H}_{i}\left(x\right)$) can be represented as:

13 $${H}_{i}\left(x\right)= {H}_{i-1}\left(x\right)+\upeta . {F}_{i}\left(x\right)$$

where ${H}_{i-1}\left(x\right)$ represents the cumulative additive output of the previous base learners, η is the learning rate controlling the contribution of each base learner, and ${F}_{i}\left(x\right)$ is the prediction of the i-th base learner. The final prediction ($Y\left(x\right)$) is obtained by summing the additive outputs of all the base learners:

14 $$Y\left(x\right)= \sum {H}_{i}\left(x\right)$$

This architecture demonstrates the iterative nature of gradient boosting, where each base learner improves upon its predecessors by focusing on residual errors, allowing gradual learning and adaptation to the data. GBM's flexibility through customizable hyperparameters, like the number of trees, learning rate, and tree depth, enables model performance optimization and addresses specific data-driven tasks^77^. GBM has proven effective in predicting and modeling sea states^78,79^.

#### Long short-term memory (LSTM)

**Figure 13:**
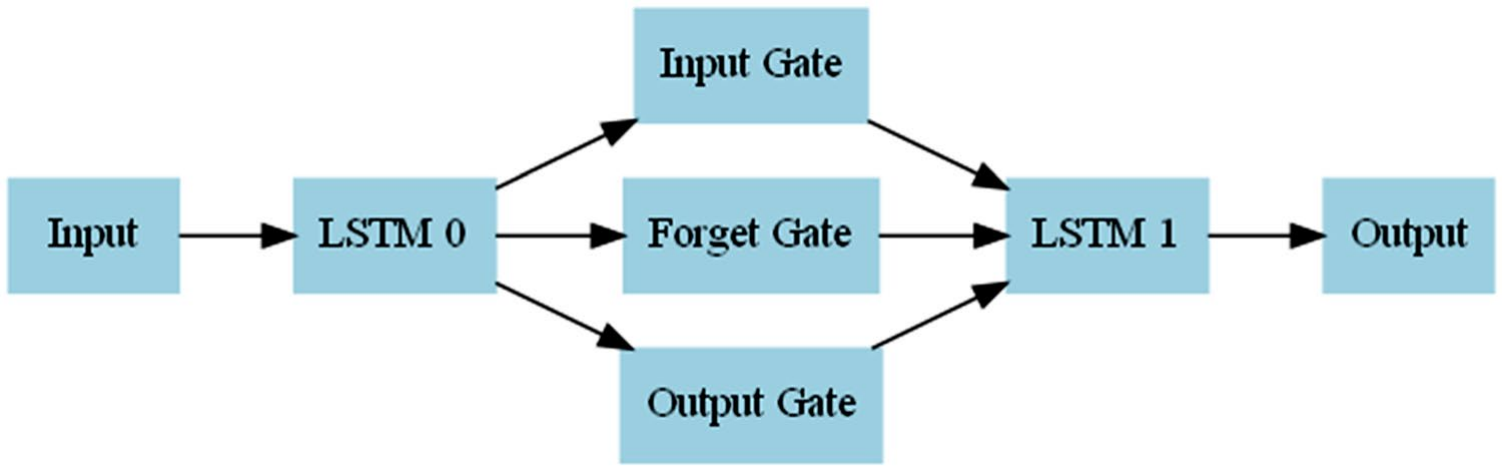
The architecture of the LSTM network shows the input, two LSTM cells (LSTM 0 and LSTM 1), and the output. Each LSTM cell includes three gates: the input gate, the forget gate, and the output gate, responsible for controlling the input, forgetting, and output of information within the cell, respectively. The LSTM network is designed to handle sequential data with the ability to capture long-term dependencies effectively.

RNNs are neural networks specifically designed to handle sequential data through recurrent connections. However, they are limited in capturing long-term dependencies due to the vanishing gradient problem, which can result in the loss of information over time. LSTM was introduced by^80^ as an improvement over traditional RNNs. It addresses the limitation of capturing long-term dependencies in sequential data by introducing specialized memory cells that allow information to persist over time. LSTM networks, depicted in Fig. 13, consist of multiple LSTM cells denoted as LSTM 0 and LSTM 1. These cells serve as the memory units of the network, capturing and storing relevant information from the input data. Each LSTM cell has three gates: the input gate, the forget gate, and the output gate. These gates regulate the flow of information within the cell, controlling the input, forgetting, and output of information, respectively. The input gate determines the incorporation of new information into the current cell state, the forget gate decides which information from the previous cell state should be discarded, and the output gate determines the amount of information passed to the next cell or the output layer. The connections between the components indicate the flow of information. The input is fed into LSTM 0, which then passes information to the input, forget, and output gates of LSTM. Finally, the output is generated from LSTM 1 and sent to the output layer. Equations (15–20) provide further details of each of the LSTM components

15 $${i}_{t}= \sigma \left({W}_{i} . \left[{h}_{t-1}, {x}_{t}\right]+{b}_{i}\right)$$

16 $${f}_{t}= \sigma \left({W}_{f} . \left[{h}_{t-1}, {x}_{t}\right]+{b}_{f}\right)$$

17 $${o}_{t}= \sigma \left({W}_{o} . \left[{h}_{t-1}, {x}_{t}\right]+{b}_{o}\right)$$

18 $$\tilde{C}_{t} = \tanh \left( {W_{c} . \left[ {h_{t - 1} , x_{t} } \right] + b_{c} } \right)$$

19 $$C_{t} = f_{t} * C_{t - 1} + i_{t} . {\tilde{\text{C}}}_{t}$$

20 $${\mathrm{h}}_{t}= {\mathrm{o}}_{t}*\mathrm{tanh}\left({C}_{t}\right)$$

where ${i}_{t}$,${f}_{t}$ ${o}_{t}$, ${\tilde{\text{C}}}_{t}$, ${C}_{t}$, and ${\mathrm{h}}_{t}$ represent the input gate, forget gate, output gate, candidate cell state, cell state, and hidden state, respectively. Similarly,$(\left[{h}_{t-1}, {x}_{t}\right])$ represents the concatenation of the previous hidden state and current input, ${C}_{t-1}$ represents the previous cell state, (${W}_{i},{W}_{f},{W}_{o},{W}_{c}$), represents the weight matrices associated with the input gate, forget gate, output gate, and candidate cell state, respectively, (${b}_{i},{b}_{f},{b}_{o},{b}_{c}$) represents the bias terms associated with the input gate, forget gate, output gate, and candidate cell state, respectively, and ($\sigma , tanh$) represents the sigmoid and hyperbolic tangent activation functions, respectively. The interconnectedness of these equations enables the LSTM to capture and store relevant information over time, addressing the vanishing gradient problem in traditional RNNs. LSTM networks have proven successful in time series data applications, such as sea state and sea level modeling and prediction^81,82^.

### Model training

In the present study, the models were trained using the filtered and detrended 13-month running mean of marine meteorological and hydrological data. These datasets comprised variables such as TSLA, HSLA, OHC, WSC, NHF, precipitation, evaporation, freshwater runoff, and atmospheric pressure. Two methods were employed: (1) the data was split into an 80:20 ratio, with 80% and 20% used for training and testing, respectively—considered a best practice in machine learning; (2) the entire dataset was used for both training and testing. The former follows best practices in machine learning models, while the latter, prone to overfitting, was performed solely to depict the temporal extent of the dataset. Grid Search hyperparameter optimization, a technique for optimizing model performance and reducing the risk of overfitting by systematically exploring different hyperparameter combinations, was employed to find the optimal combination of hyperparameters for the models. The models were then instantiated, and the training data was fed into them to capture the underlying patterns and relationships between the variables.

### Model performance evaluation

Moving forward, we present the model prediction performance evaluation metrics used in the present work to assess the reliability and accuracy of the models in predicting the MSLA in the GoG. The coefficient of determination (${R}^{2}$), a metric that assesses the proportion of the total variance in the observed data that can be explained by the model predictions, and the root mean square error (RMSE), a metric for assessing the model's predictive skill, were employed. As a standard model evaluation approach, the R^2^ value ranges between 0 and 1. A value of 0 indicates that the model does not explain any variability in the data, indicating poor performance. Conversely, a value of 1 signifies that the model perfectly explains all the variability in the data, indicating better performance. However, a lower RMSE value corresponds to higher prediction accuracy and better model performance. Conversely, a higher RMSE value suggests lower accuracy and poorer model performance. The two evaluation metrics are expressed as follows:

21 $$R^{2} = 1 - \frac{{\mathop \sum \nolimits_{i = 1}^{N} \left( {Y_{i} - X_{i} } \right)^{2} }}{{\mathop \sum \nolimits_{i = 1}^{N} \left( {Y_{i} - {\tilde{\text{Y}}}} \right)^{2} }}$$

22 $$RMSE= \sqrt{\frac{1}{N}\sum_{n=1}^{N}{\left({X}_{i}-{Y}_{i}\right)}^{2}}$$

where N is the number of data points in the sample, ${Y}_{i}$ represents the observed values of the dependent variable, ${X}_{i}$ represents the predicted values of the dependent variable based on the regression model, and ${\tilde{\text{Y}}}$ represents the mean of the observed values of the dependent variable.

## Data Availability

The datasets analyzed during the current study are: sea surface height (SSH) hereafter referred to as mean sea level anomaly (MSLA), sea surface temperature (SST), sea surface salinity (SSS) data provided by the Copernicus Marine Environment Management Services (CMEMS), available at http://marine.copernicus.eu/product. Additionally, air temperature, u wind (10 m), v wind (10 m), Total precipitation, Evaporation, Atmospheric pressure, Net Shortwave Radiation , Net Longwave Radiation, Surface Latent Heat Flux, and Sensible Heat Flux at a single pressure level provided by the European Centre for Medium-range Weather Forecasts (ECMWF, 2011) reanalysis era5 data, available at http://cds.climate.copernicus.eu/.
